# Diabetes, Mellites and Insipidus

**Published:** 1890-03

**Authors:** 


### "DIABETES, MELLITUS AND INSIPIDUS."

BY ANDREW H. SMITH, M. D., "THE PHYSICIAN'S LEISURE LIBRARY" SERIES, GEORGE T. DAVIS, PUBLISHER, DETROIT, MICH.

This excellent little work will be found a most satisfactory exposition of the practical views and facts concerning these diseases, according to the present status of medical knowledge. Thoroughly practical, the eminent author has admirably carried out the object of this little work, as expressed in his modest preface, where he says that he does not aim "to compress into the fewest possible words all that is known or surmised in regard to diabetes, but to give points which will most interest those who have to manage cases of this disease." His style is clear, terse, and interesting; and shows the practical physician throughout. The clinical history and complications of the disease is well brought out. Uuder "Causes, pathology and morbid anatomy," he presents in a succinct form the best accepted views upon subjects, concerning which there yet remains much to be learned. Under "Diagnosis" he gives the best and simplest formulæ for testing the urine. The chapter on "Treatment" is very full, and some valuable formulæ are given, together with a notice of those remedial agents which have best stood the test of experience. Some of the most recently used drugs, which promise good results, are particularly referred to, and in this connection, the remarkable effects of antipyrin, in the hands of Huchard, Robin, Germain Lee, and such reliable observers, is quoted almost entire from the article in the "Theapeutic Gazette," so important does our author consider the claims therein set forth. Also upon the same subject, a resume is given of the recent discussion in the Academie de Medicine, (Paris), which devoted an entire session to the consideration of antipryin as a remedy in diabetes. Dr. Smith clearly reflects in these pages, the results of his own experience, and while he disclaims originally for his little work which, he remarks, "must, from the nature of the case, be largely a compilation," yet we have no hesitation in saying that it is a valuable "compilation," and as such we cheerfully commend it to the "practitioner," busy or otherwise. The book is printed in good, clear type, upon excellent tinted paper. It is bound in a substantial, attractive paper binding, and furnished for the moderate sum of twenty-five cents; in cloth for fifty cents. The excellent "series" of which this is one volume, certainly places good medical literature within the reach of all. J. C. O.

